# Evolvability in vertebrate segmentation

**DOI:** 10.1016/j.semcdb.2025.103630

**Published:** 2025-10

**Authors:** James E. Hammond, Callum V. Bucklow, Berta Verd

**Affiliations:** Department of Biology, University of Oxford, Oxford, UK

**Keywords:** Evolvability, Evolution, Development, Somitogenesis, Vertebral counts, Evodevo

## Abstract

The number of vertebrae in the axial skeleton of vertebrates is extremely diverse, and reflects adaptations to a diverse range of habitats and lifestyles. The capacity for heritable evolutionary change in the number of vertebrae — its evolvability — is underpinned by the process of somitogenesis, which determines the number of somites that form in the early embryo. However, despite the evolvability of somitogenesis having been crucial for the success of the vertebrates across evolutionary history, the developmental sources of evolvability in somitogenesis are still unknown. Here, we review the evolution of somitogenesis and vertebral number, and attempt to identify sources of evolvability within this important developmental process.

## 1 Introduction

The body plans of vertebrates are extremely diverse [1], [2], [3], [4], [5], [6]. A major contributor to this diversity is the number of vertebrae in the axial skeleton, which can range from as few as 7 in the diminutive terrestrial frogs in the genus *Paedophryne* [7], to more than 750 in deep-sea *Nemichthys* Snipe eels [4], [8], [9]. The capacity for variation or ‘evolvability’ in the number of vertebrae varies between clades, for instance in some groups this variation is low and the number can be taxonomically definitive [4] while in others, vertebral number can be highly variable, such as the Ophidiidae Cusk-eels which vary from 58 to 91 vertebrae in total [8] or the African cichlids (Pseudocrenilabrinae) which vary from 25 to 40 vertebrae [10], [11], [12]. Evolvability of this trait has been a major driver of vertebrate evolution, as changes in vertebral number have enabled vertebrates to colonise new niches throughout evolutionary history [13], [14], [15]. However, the developmental origins of this evolvability remain elusive and poorly understood.

The development of the vertebrae is a well-characterised process, where the number of vertebrae is specified in early development by the number of somites [16], [17], paired blocks of mesoderm that segment the developing trunk body, and give rise to the skeletal muscle and axial skeleton [16]. Somitogenesis is a highly dynamic process, with somites arising sequentially from an unsegmented region of mesoderm at the posterior of the developing embryo, known as the pre-somitic mesoderm (PSM). The process by which somite boundaries are pre-patterned in the PSM is well-characterised, and is thought to be driven by a complex molecular oscillator known as the ‘segmentation clock’ that drives travelling waves of synchronised gene expression from the PSM posterior to the anterior [18], [19], [20]. The total number of somites formed is thought to be inversely proportional to the frequency of the segmentation clock [18], [21], [22], and the dynamics of the clock are known to vary across species [23], [24], for instance in the Zebrafish (*Danio rerio*) the clock oscillates approximately once every 30 min [20], [25], every 90 min in the Chicken (*Gallus gallus*) [19], every 120 min in the Mouse (*Mus musculus*) [26], or 320 min in Humans [26], suggesting that evolution of the clock may be involved in generating the diversity of vertebral number. While we are now beginning to understand the processes that give rise to such a diversity of dynamics [24], [26], the developmental sources of evolvability in somitogenesis remain elusive.

Understanding evolvability, the capacity of a biological systems to evolve heritable and adaptive phenotypes, and its relationship to the mapping between genotype and phenotype, is a major research goal within evolutionary developmental biology (‘evo-devo’) [27], [28], [29], [30], [31]. While the term ‘evolvability’ is widely credited to Dawkins (1989) [32], who studied whether a lineage’s capacity to evolve and adapt under natural selection could itself evolve using *in silico* life forms, the study of how properties of development may affect the trajectory of evolution is itself older [27], [28], [33], [34]. Evolvability is thought to be determined by a variety of processes, such as the rates of mutation and recombination of genetic material [35], phenotypic plasticity in response to environmental factors [36], stochastic gene expression and resulting phenotypic heterogeneity [37], pleiotropy [38], canalisation and robustness of the genotype-phenotype map [39], [40], the complexity of the genotype-phenotype map [34], [41], and the way in which adaptive or fit phenotypes are selected by evolution [42], [43].

While much of the work on evolvability has been theoretical, there now exists a large corpus of experimental studies investigating the causes of evolvability and their evolution (reviewed in [44]). Experiments involving techniques suited to microorganisms, such as experimental mutagenesis [45], [46], laboratory studies of evolution [43], [47], [48], [49] or design of synthetic gene regulatory networks [37], [50] have proven extremely powerful in furthering our understanding of evolvability. However outside of a handful of examples [51], [52], [53], there are relatively few experimental or data-driven studies of the developmental bases of evolvability in multicellular systems. This is in part due to the experimental challenges involved in performing comparative studies of development between closely related taxa, and also because in order to determine the causality of development in evolvability, realistic and accurate data-driven mathematical models are required [27], [28], [33]. With this in mind, the study of vertebral number in vertebrates provides an excellent model system for understanding the developmental sources of evolvability as there exists a rich repertoire of data-driven mathematical models describing the segmentation clock [54], [55], [56], [57], [58], [59] and an increasingly large body of comparative data [23], [24]. Here, we examine the evolvability of vertebral number in vertebrates and discuss possible sources of evolvability within somitogenesis.

## 2 Evolvability of segment number

**Fig. 1:**
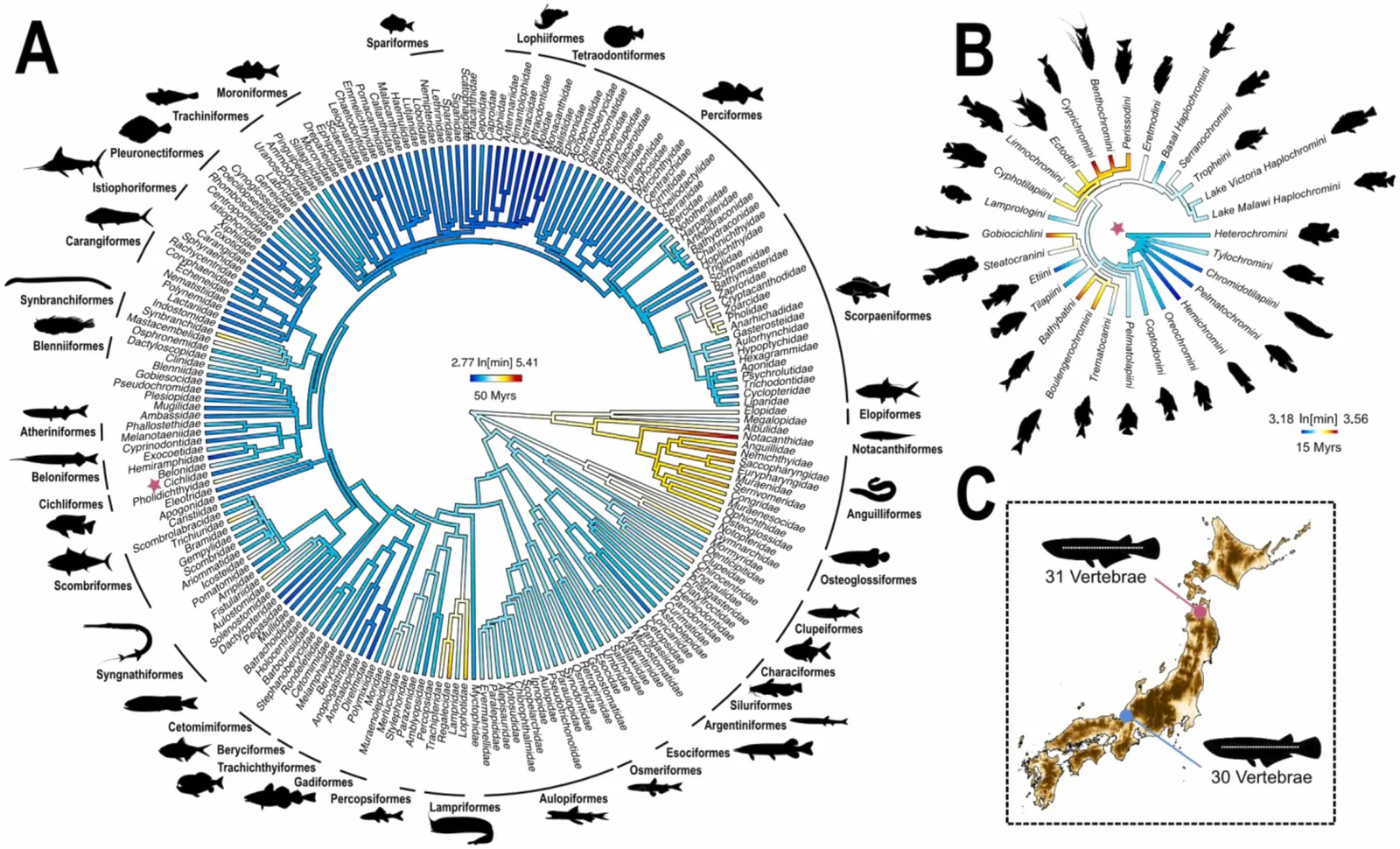
Diversity in vertebral number across the ray-finned fishes. **A** Family-level phylogeny of the ray-finned fishes (Actinopterygii) after Betancur *et al.*[68], with tips coloured according to the log-transformed minimum number of vertebrae reported for that family by Nelson *et al.*[4]. Orders are annotated, with silhouettes of exemplar species. Silhouettes were downloaded from PhyloPic (https://www.phylopic.org/). Image credits and licenses: Emily Troyer (CC BY 4.0), Erika Schumacher (CC BY-NC 3.0), U.S. Fish and Wildlife Service (illustration) and Timothy J. Bartley (silhouette) (CC BY-SA 3.0), Tree of Life App (CC BY 4.0), Mykle Hoban (CC BY-SA 3.0), Grupo de Ictiología de la Universidad de Antioquia (CC BY 4.0), and Richard Winterbottom and Russell Engelman (CC BY 4.0). **B** Tribe-level phylogeny of the african Cichlidae, after [69]. Tips coloured according to the log-transformed minimum number of vertebrae from the corresponding tribe [11], [12]. Silhouettes were downloaded from PhyloPic or created in Inkscape. **C** Different populations of japanese Medaka (*Oryzias latipes*) show heritable differences in vertebral number ([61]).

The number of vertebrae in the vertebrate axial skeleton exhibits a high degree of evolvability, as can be seen in the ray-finned fish (Actinopterygii) (Fig. 1A). Here, we see repeated independent transitions between low and high vertebral number at a family-level resolution, as evidenced by examining the phylogeny of this clade (Fig. 1A).The same pattern of repeated independent transitions in vertebral number is also present within clades [8], [60], and at the infra-family scale, e.g. within African cichlids (Cichlidae, Pseudocrenilabrinae) (Fig. 1B) [10], [11]. Vertebral number is also known to vary between populations of the same species, e.g. in the Medaka *Oryzias latipes* which ranges from 30 to 31 vertebrae [61] (Fig. 1C), the Arctic Charr *Salvelinus alpinus* (58−64) [62], the Kōaro *Galaxias brevipinnis* (57−62) [63], the Northern pike *Esox lucius* (56−63) [64], or the Deer mouse *Peromyscus maniculatus* (23–27 caudal vertebrae) [15]. While some of this variation may be attributable to environmentally driven developmental plasticity [64], [65], in at least the Pike, Medaka, and Deer mouse such variation has been shown to be heritable [15], [61], [64]. It therefore appears that heritable changes in vertebral number can occur under presumably minor or at least evolutionarily rapid changes to the genotype, and thus that vertebral number exhibits a high degree of evolvability.

Vertebral number is also an adaptive trait: longer tails with more vertebrae permit forest populations of Deer mice to climb better than their prairie-dwelling congeners [15]. Deep sea fish show typically elongate eel-like bodies, thought to be an adaptation for energy-efficient anguilliform swimming under increased hydrostatic pressure [13]. In some cases, this is associated with extreme increases in the number of vertebrae [9]. Similarly, fossorial snakes possess smaller vertebrae relative to their body than fast-moving terrestrial or arboreal species [1], which could in principle arise via an increase in the number of vertebrae while reducing the length of the vertebrae to preserve overall body length but allowing greater flexibility, as may possibly be required for such a lifestyle. In a similar way, fast predatory fish such as Barracuda or Scombriformes are known to have relatively few vertebrae compared with other similarly elongate fish [66], which may stiffen their body axis, giving better propulsion when pursuing prey [67].

## 3 Development of vertebral number and its evolution

The number of vertebrae in the adult vertebrate is determined by the number of somites that form in the early embryo [16], [17]. Somites are paired transient blocks of mesoderm that segment the trunk anterior-posterior axis, and determine the future segmentation of the body’s musculature, nervous system, and axial skeleton [16]. The number of somites is partially controlled by the frequency of an intracellular oscillator known as the segmentation clock, which synchronises differentiation of cells in the pre-somitic mesoderm (PSM) and thus controls the timing of the regular budding of cells out of the PSM to form the somites [18], [19], [21].

The action of the clock is most often explained in terms of the clock and wavefront model [18], [70]. In brief, this model assumes that the phase of intracellular oscillations in gene expression are ‘read-out’ by development at a point in space known as the wavefront. This wavefront moves posteriorly as the embryo elongates, and thus the temporal information from the oscillator can be read out into a spatially periodic pattern [18]. The identity and nature of the wavefront is controversial but is generally thought to be under the control of FGF signalling [21], [70], [71], [72], [73], most likely via ERK signalling [74], [75], [76]. The coupling of this wavefront to elongation of the embryo is thought to be, at least in part, due to the progressive decay of FGF mRNA in PSM cells, creating a gradient of positional information [77] from the posterior to the anterior. The identity of clock components is less controversial and there is strong evidence that the clock is driven by auto-repressive feedback of genes in the Hes/Her family [19], [54], [78], [79], [80], [81]. Intracellular oscillations of Hes/Her transcription factors are noisy [82], [83], are synchronised by delta notch signalling [55], [81], [82], [84], and decrease in frequency towards the PSM anterior [85], creating travelling waves of gene expression which travel along the PSM towards the anterior [20], [82].

The frequency of the clock thus determines the total number of somites (and therefore vertebrae) that can be formed [21], [22], [86]. However, the clock and wavefront model also implies that the duration of somitogenesis is causal in determining the number of vertebrae in the body. Developmental control of the duration of somitogenesis is less well-understood, and it is thought to depend both on gene regulatory and morphogenetic processes [15], [21], [87], [88]. For one, the total number of somites in the embryo correlates with how much the PSM elongates over the course of somitogenesis [23], [88], which has been explained by termination of somitogenesis being induced by a threshold value of retinoic acid (RA), an anterior-posterior decreasing gradient which is secreted from the somites, and thus somitogenesis terminating when the PSM is sufficiently short [21], [89]. In this way, it is thought that maintenance of PSM length over time by morphogenesis determines the duration of somitogenesis, and with it, the total number of vertebrae formed. What controls the maintenance of the PSM over the course of somitogenesis is unclear, however the point in time at which PSM elongation halts has been shown to correlate with the expression of Hox13 genes [90], [91], [92], [93], [94], [95], and temporal changes in Hox13 expression have been implicated in evolutionary changes in vertebral number in reptiles and mice [15], [91].

**Fig. 2:**
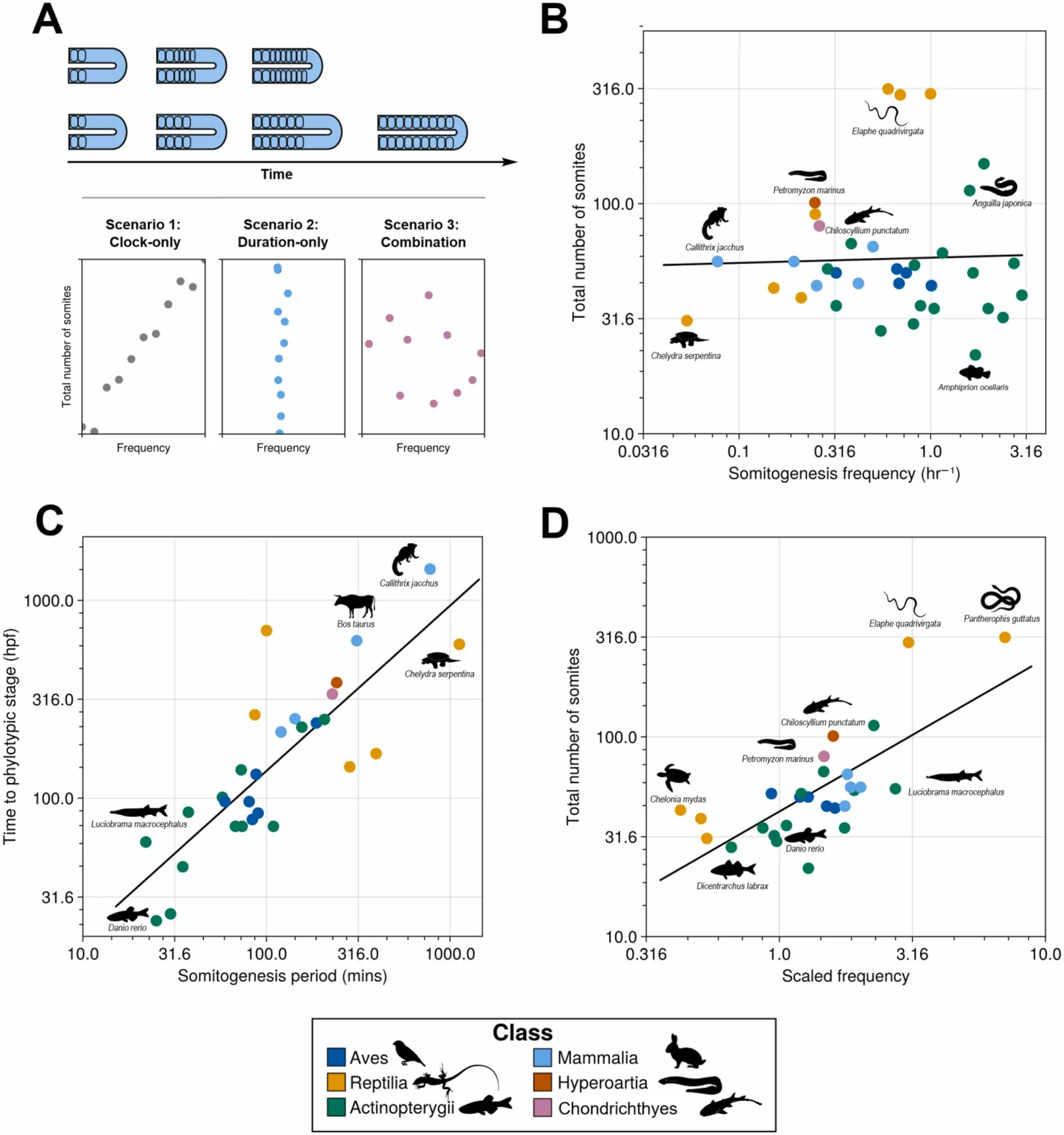
Diversity in somitogenesis dynamics across the vertebrates. **A Top:** the same number of somites (black rectangles) can be achieved by changes in either frequency (top) or duration (bottom) of somitogenesis. **Bottom:** Scenarios for plotting the total number of somites formed during embryogenesis against somitogenesis frequency. If diversity is due to only evolutionary changes in frequency, the datapoints should lie along a linear trendline (left, black). Conversely if diversity is due to only changes in duration, the datapoints should lie along a vertical line (middle, blue). If diversity is created by a combination of the two, then the datapoints are heterogeneously spread across the space (right, mauve). **B** The total number of somites formed during embryogenesis, plotted against the frequency at which somites are formed. A linear trendline is fitted from regression of the log-transformed data, with trendline *y = 0.023x −1.765* with the summary statistics *p = 0.8423, R*^*2*^*= 0.001*, suggesting that there is no correlation between the dependent and independent variables. **C** The time taken to reach phylotypic stage (defined as presence of four pharyngeal arches), plotted against the somitogenesis period. A linear trendline is fitted from regression of the log-transformed data, with trendline *y = 0.835x −0.467* with the summary statistics *p < < 0.01, R*^*2*^*= 0.674*, suggesting a statistically significant relationship. Silhouettes were hand-drawn or downloaded from Phylopic (https://www.phylopic.org). Image credits and licenses: Edwin Price CC BY 4.0 and DFoidl and T. Michael Keesey CC BY-SA 3.0. Code for generating the figures, and raw data, can be found in the supplementary material.

It is however unclear from the current literature whether one of frequency or duration is most often employed by evolution to alter an organism’s vertebral number, or if one is more evolvable. To explore the relative importance of changes to clock frequency or duration of somitogenesis in evolution, we performed a literature survey, cataloguing the number of somites formed during embryogenesis and the rate of their appearance in different vertebrate species (see supplementary table 1). We can imagine three scenarios for this data (Fig. 2A). In scenario one, diversity in somite number is solely explained by evolution of the clock frequency, and we would expect to see a positive correlation between somite number and frequency, with the datapoints tightly distributed along the trendline (Fig. 2A, left). In scenario two, where diversity in somite number can be explained by evolution of the duration of somitogenesis alone, we would expect to see the datapoints lie along a vertical line when we plot the number of somites against the clock frequency (Fig. 2A). The third scenario is that the diversity in segment number is due to co-evolution of these two processes, and we would expect to see the datapoints spread along each axis (Fig. 2A).

Plotting the total number of somites formed in the embryo against the rate of somitogenesis (see supplementary material for methods and data), we see no clear trend, and that the frequency of somitogenesis and the total number of somites is extremely diverse across different groups of vertebrates (Fig. 2B). Within our dataset, the slowest rate of somite formation is the Common snapping turtle (*Chelydra serpentina*), with one somite being formed every 19 h [96], and the quickest is the Mexican tetra (*Astyanax mexicanus*), with one somite being formed every 20 min [97]. However, for appropriate comparison between species the rate of somitogenesis must be scaled against the overall pace of development, which varies across taxa [24], [26]. It should be noted that the frequency of somitogenesis is highly temperature-sensitive [25], [98], and so one must also account for differences in the temperature of development between species. To do so we assume that the frequency of somitogenesis and the overall pace of development scale together in response to changes in temperature, and so hope to implicitly normalise the data for varying temperatures by normalising against the overall pace of development. We see that the period of somitogenesis (i.e., the time taken to form one somite) correlates well with the time taken for the embryo to reach the phylotypic stage (defined by the first appearance of the pharyngeal arches), and the slow frequency of somite formation in species like the Common snapping turtle or the Common marmoset (*Callithrix jacchus*) can largely be explained by their overall slower pace of development (Fig. 2C). This is similar to the results of [24] who observe that the period of the segmentation clock *in vitro* scales with the overall pace of development [24]. Scaling the frequency against this overall rate of development (see methods) and plotting against the number of somites reveals that the total number of somites correlates with frequency, but that the data is heterogenous. This suggests that while evolution of the frequency of somitogenesis has been a major driver of the evolution of vertebral number, it cannot explain all of the extant diversity in vertebral number, and the data is best explained by scenario three (Fig. 2D).

Here we see that some species, such as the Japanese striped snake *Elaphe quadrivirgata* or the Corn Snake *Pantherophis guttatus* exhibit accelerated clocks and their large numbers of somites can be at least partially attributed to this fact (Fig. 2D) [23]. We also see that some active predatory fish such as the European seabass (*Dicentrarchus labrax*) form few somites and have correspondingly slow clocks (Fig. 2D), suggesting that perhaps this reflects selection for a stiffer vertebral column with fewer vertebrae [66], [67]. However, some species such as the Long spiky-head carp (*Luciobrama macrocephalus*) possess rapid clocks, approaching the snakes’ in their frequency, but do not form as many somites (Fig. 2D). This suggests that the duration of somitogenesis is shorter relative to the frequency of the clock in the Long spiky-head carp than in snakes and highlights that even in cases where the clock is dramatically accelerated, evolution of duration cannot be discounted when considering the evolution of somite number [23].

We also note, as reported by [24], that the species of mammals analysed here have roughly equivalent somitogenesis frequencies when one accounts for differences in developmental rate (Fig. 2D), suggesting that perhaps differences in mammalian vertebral number are not driven by changes in the clock but rather differences in the duration of somitogenesis [15]. The evolution of the mammalian vertebral column is thought to be highly constrained by its regionalisation, with number of pre-sacral vertebrae being highly conserved, likely reflecting mechanical constraints imposed by the running gait of most mammals [28], [99]. It is possible therefore that this invariance in scaled clock frequency reflects a constraint on the pace of the clock relative to the pace of the ‘Hox timer’ or the sequential activation of Hox genes in the embryo [100] (assuming such a timer also scales with the overall pace of development), thereby ensuring a constant number of pre-sacral vertebrae. While this is speculative, the accessibility of non-model mammalian species for developmental biology is rapidly improving [24], and it may soon be possible to verify if the developmentally-adjusted frequency of the clock is indeed a conserved feature of mammals.

This analysis also reveals that when the overall pace of development is accounted for, the Testudines (turtles, terrapins, and tortoises) have a very slow rate of somite formation (Fig. 2D). The three species of Testudines in our dataset, the Common snapping turtle, the Green sea turtle (*Chelonia mydas*), and the Red-bellied short-necked turtle (*Emydura subglobosa*), all have similarly low frequencies of somite formation (Fig. 2D), suggesting this is possibly an ancestral feature of the Testudines, as the lineages leading to *C.mydas* and the other two species diverged at the base of the testudines clade [101]. The functionality of this is unclear, but perhaps could reflect an adaptation for decreased vertebral number and increased rigidity of the axial skeleton, which is thought to predate the evolution of the Testudine shell [3], [102]. Study of the Testudine segmentation clock may prove to be illuminating for understanding the evolvability of vertebral number, particularly in terms of understanding the clock’s capacity for change in both dynamics and regulatory architecture, as well as a comparison with snakes, which appear to have taken the diametrically opposite path in evolution (Fig. 2D).

We can also observe that the frequency of somite formation is approximately the same in the Sea lamprey (*Petromyzon marinus*) and the Brown banded bamboo shark (*Chiloscyllium punctatum*) (Fig. 2D). Whether this is coincidental or not remains to be seen, however we consider it of note due to the position of both species at the base of the vertebrate clade [103], [104]. If such a trend were to hold more generally and the developmentally-normalised rate of somitogenesis is conserved between the cyclostomes and elasmobranchs it would suggest that the extant evolvability of the clock arose only later in vertebrate evolution. Data on somitogenesis is currently extremely limited within these clades and sparse in general, but as more data becomes available we suggest that the field takes note of any conservation of somitogenesis frequency between these two groups.

Overall, this analysis suggests that both the morphogenesis of the PSM and the clock co-evolve to generate diversity in segment number. We cannot identify from this dataset whether or not one process is more labile under evolution than the other, and both processes appear to have contributed to significant evolutionary changes in vertebral number. To identify one process is truly more labile or typically occurs first in evolution, we suggest that future work should focus on comparative developmental studies of closely related species, to understand the changes that occur over such short evolutionary evolutionary timescales.

## 4 Developmental sources of evolvability

While the segmentation clock is thought to be driven by auto-repressive feedback on Hes/Her gene expression, the clock contains a large number of oscillatory and non-oscillatory components [105], [106]. It is possible that this has been a rich substrate for evolution. The formation of non DNA-binding Hes/Her dimers is known to accelerate clock oscillations [22], [79], and therefore duplication of Hes/Her family genes and their expression in the PSM could potentially result in changes to the frequency of the clock. The Hes/Her family contains many paralogs within the vertebrates [107], and Hes/Her family genes have been shown to have undergone repeated duplications across the metazoa [108], [109], [110], [111], suggesting that perhaps the appearance of novel paralogs or the neo- or sub-functionalisation of paralogous genes in this rapidly-duplicating family may be a major source of evolvability for clock dynamics. Indeed, the identity of Hes/Her oscillatory genes in the clock can vary between species of teleosts [108], suggesting a role for neo-functionalisation. Similarly, altering the copy number of delta genes has been shown to increase the emergent frequency of the clock as a result of elevated clock coupling between cells [112]. Lastly, the presence of paralogs can also create redundancy within the clock, such as in the case of the *her1* and *her7* genes in zebrafish [79]. It is possible that such redundancy has allowed neutral exploration of genotypes in evolution, allowing the discovery of novel, highly divergent clock dynamics and expression [39]. We can expect the role of gene duplication in the evolvability of somitogenesis to become clearer as the availability of comparative data increases.

It is possible that a similar property exists for the morphogenetic processes underpinning elongation of the PSM. Across different vertebrate species elongation of the PSM is known to be driven by cell division [88], as well as ingression of nascent PSM progenitor cells from surrounding tissues [88], [113], [114], dynamic changes in cell motility [114], [115], [116], [117], and cell density [115], [118], [119]. The degree to which anyone of these processes contributes to elongation of the PSM appears to vary across species [23], [88], which could suggest that diverse PSM elongation dynamics can be generated by the combinatorial effects of these processes. If this were the case, then the existence of a set of uncoupled morphogenetic processes such as these could be regarded as a source of evolvability for somitogenesis, however whether such processes are uncoupled is as yet deeply unclear and the development of cell-based models of PSM elongation [59], [115], [118], [120], [121] will reveal whether this is the case, and the scope for neutral exploration of the mechanisms underlying PSM elongation by evolution.

The topology of the segmentation clock gene regulatory network may also be a source of evolvability, specifically the existence of two oscillatory circuits: one being the Hes/Her auto-repressive loop, and the other the negative feedback of delta-notch signalling onto itself via intercellular signalling [54]. Using a simple model, Lewis [54] demonstrated that the relatively long time delay created by intercellular signalling allows oscillations with much lower frequency to be created when auto-repression of Hes/Her expression is decreased, and speculated that the covariation in these two processes could perhaps give rise to different clock frequencies in evolution [54]. However, simulations suggest the existence of a parameter space with irregular oscillations as the relative contribution of delta/notch to the circuit increases, and thus the evolution of this system may actually be constrained in this regard [54]. Inferring the relative contribution of each circuit in determining the clock’s frequency is a hard problem as it requires experimental parametrisation of this model, and to our knowledge there have not been comparative studies examining the contributions of each circuit nor their change in evolution.

The delay in intercellular communication via delta-notch is also likely to exert constraints on the evolution of the clock gene regulatory network, and vice versa. In order for stable and synchronous oscillations to be formed the delay in intercellular signalling must satisfy a relationship with the frequency of auto-repressive intracellular oscillations of Hes/Her genes where signals are sent and received by cells in the correct phase of the clock [54], [122]. The delay in intercellular signalling is thought in part to be controlled by the number of intermediary molecules, such as lunatic fringe (Lfng), involved in the notch signal transduction pathway [123], and therefore the number of intermediary components in signal transduction may be constrained by the frequency of Hes/Her oscillations. We also can expect the converse to be true, and that the frequency of Hes/Her oscillations is constrained by the existing signalling delay. However, the collective frequency of oscillations is also set by the value of the delay [54], [122], and so it may be that evolutionarily neutral regions of parameter space exist where the delay and the frequency may co-vary without perturbing the overall frequency. Only with further theoretical study will it become clear to what capacity the delay and frequency of the clock mutually constrain one another’s evolution.

It is unclear how the behaviour of the oscillatory components of the FGF/Wnt pathways [105], [106], [124], [125], which are capable of changing their expression dynamics in evolution [106] might affect segmentation clock dynamics. Indeed, in Mouse at least it appears that the dynamics of Wnt and Notch signalling are coupled, with functional significance for somite polarity [125]. However, there is limited comparative data on whether this is a conserved feature of vertebrates and as such we cannot predict in what way regulation may affect the evolution of the clock. Oscillations in ERK signalling, a component of the FGF signalling pathway, are significant for the differentiation of PSM cells into somites [76], [126], and have been suggested to control the slowing dynamics of the clock towards the anterior of the PSM [126]. Indeed, FGF signalling appears to be required for sustained oscillations of Hes/Her genes [83], [127], and in Zebrafish FGF signalling controls the expression of the frequency modulating transcription factor Hes6 [79], [128]. Additionally, we note that as PSM morphogenesis is itself under the regulation of Wnt and FGF signalling [76], [113], [115], [116], [117], the evolution of morphogenesis and the dynamics of the clock may be prone to co-vary according to changes in Wnt or FGF signalling.

Vertebral number has been shown to also depend on maternal effects [129], [130], as have the period of somitogenesis and its duration [129]. The total duration of somitogenesis has also been shown to correlate with the amount of yolk available to the embryo [88], and the number of somites been shown to be lowered in conjoined twin embryos of the Lake trout *Salvelinus namaycush*, leading to a suggestion that the number of vertebrae depends on the quantity of yolk available to the embryo [131]. Maternal contribution to the offspring can vary with the age of the mother [132], suggesting this as a possible source of phenotypic heterogeneity in otherwise genetically homogenous populations, and thus a source of evolvability in vertebral number. However it is unknown whether age-specific effects occur in the vertebral number of offspring.

Vertebral number is also known to exhibit environmentally-induced phenotypic plasticity, with vertebral number responding to changes in salinity, light, and temperature [64], [65], [133]. Due to their external mode of development, this has been most extensively studied in the teleost fishes. The environmental effect of temperature here is perhaps counter-intuitive, as the frequency of somitogenesis increases with increasing temperature [25], [98], [134], whereas it is typically observed in nature that lower temperatures yield fish with more vertebrae [61], [64], [65], [135]. Indeed the relationship between embryo temperature and vertebral number is thought to be U-shaped, where the maximal number of vertebrae occurs at extremely low or extremely high temperatures and at intermediary temperatures the lowest number of vertebrae are formed [133], [136]. The developmental basis of such a trend is unclear. Furthermore, the effect of temperature on somitogenesis is thought to be buffered by the invariance of somite length [25], likely due to a scaling in wavefront and clock molecular kinetics [137]. It is likely therefore that this scaling relationship, or any scaling relationship that exists between the frequency and duration of somitogenesis, breaks down at extreme temperatures. While it is probable that the plasticity of vertebral number has facilitated the rapid evolution of vertebral number, to our knowledge this has not yet been studied, and it is unclear if this indeed the case.

While our understanding of somitogenesis and the segmentation clock has benefitted from a large body of theoretical work, it is still difficult to definitively identify sources of evolvability within somitogenesis. This is in large part due to a still incomplete understanding of the gene regulatory architecture underpinning the segmentation clock and the wavefront, as well as the computationally complex problem of understanding how cellular behaviours such as motility and division give rise to the elongation of the PSM, and the added difficulty of parameterising such models using experimental data. Furthermore, comparative studies of somitogenesis have largely been limited to vertebrate model species that are significantly diverged [23], [106], [108], [109], and there have been few comparative studies examining how vertebral number and the components and dynamics of somitogenesis co-vary over short evolutionary timescales [15], [138], so it is difficult to suggest from observational data where the sources of evolvability may lie.

## 5 Modularity of the segmentation clock and PSM elongation

Modularity, the dissociability of developmental processes and pathways by evolution, is thought to be a major source of evolvability across the tree of life [29], [139], though evolvable systems can also exist without modular organisation [140]. The evolvability of diverse phenotypes such as butterfly eyespots [141], beak shape in the Galápagos finches [142], arthropod limbs [143], [144], arthropod segmentation [52], and the regionalisation of the vertebrate skeleton [8], [100], is thought to be underpinned by modularity of development.

In a recent study, we predicted that the clock and morphogenesis of the PSM exhibit modularity in zebrafish, and thus, that this might heighten the evolvability of vertebral number [120]. We predicted that the modularity is dependent on properties of morphogenesis such as tissue length and density, but more so on properties of the clock such as the strength of delta-notch signalling and the coupling delay [120]. Due to a lack of comparative experimental data we were only able to predict that this is true in zebrafish, however we see no reason to expect why this property is restricted to zebrafish and not a conserved feature of the vertebrates, particularly in light of the apparently combinatorial effects of somitogenesis pace and duration in driving diversity of vertebral number (Fig. 2).

Understanding the selective or neutral ways in which modularity arises in evolution is an open question in evolutionary biology [29], [31], [145]. To our knowledge somitogenesis is one of the few developmental systems where modularity has been predicted to depend on such a small set of experimentally tractable parameters, and thus we suggest that a quantitative study of the evolution of the segmentation clock and the PSM may be extremely insightful for this question.

## 6 Conclusions and future work

While much is known about somitogenesis and the developmental control of vertebral number, the developmental basis of its evolvability is still unclear. However, many of the properties of the segmentation clock and PSM morphogenesis can be placed in the context of previous theoretical work to predict possible sources of evolvability. The reasons for this lack of understanding are twofold, and reflect a lack of comparative studies with closely-related species, as well as of experimentally parameterised models of somitogenesis in different species. We note that technological advances are increasingly allowing the field to undertake comparative studies of somitogenesis [24], and suggest that these technologies be employed to study closely related groups of organisms to understand the relative lability of the various components of somitogenesis in evolution. We also suggest that the study of the reptiles, particularly a comparative study of the Serpentes and Testudines, could be illuminating in understanding the extreme limits of somitogenesis and elucidate which changes to somitogenesis can generate extreme diversity.

## 7 Methods

Mapping of vertebral number to phylogenies was completed in R (v4.2.0) [146]. Ranges for 271 Teleostean orders and families were collated primarily from Nelson’s ‘Fishes of the World’ [4] and for the tribes and subtribes of Pseudocrenilabrinae from previously reported data [10], [11]. We pruned the time-calibrated, bony fishes phylogeny published by [68] to the families present within our vertebral counts dataset in the R package, ape (v5.7.1) [147]. To reduce each family to a single branch and to account for the estimated age of each respective family, the tree was pruned to the longest (i.e. most basal) branch within each family, an approach we utilised again to prune the Pseudocrenilabrinae phylogeny published by [69] to each respective tribe and subtribe. Log-transformed minimum total vertebral and ancestral states were mapped and inferred using the function contMap, part of the phytools (v2.1.1) package [148]. R code for generating the figures can be found in the supplementary file *mapping_vertebral_counts.R*^.^

The data in Fig. 2 reflects either reported values for the period and frequency of somitogenesis, or values that have been inferred by linear regression of the reported number of somites over time (see supplementary table 1). Linear regressions between frequency and the number of somites, or period and the time to phylotypic stage, were performed in Julia v1.8.2 using the function GLM.lm. Julia code for generating the figures can be found in the supplementary file *somitogenesis_pace.jl*.

## Declaration of Competing Interest

The authors declare the following financial interests/personal relationships which may be considered as potential competing interests: Berta Verd reports financial support was provided by a 10.13039/501100000781European Research Council Starting Grant 101163722/COUNTS. Callum V. Bucklow reports financial support was provided by a 10.13039/501100000268Biotechnology and Biological Sciences Research Council Studentship (grnat number 2445747). James E. Hammond reports financial support was provided by a UK Research and Innovation Natural Environment Research Council DTP Studentship (grant number: NE/S007474/1). The authors declare that they have no known competing financial interests or personal relationships that could have appeared to influence the work reported in this paper.

## Footnotes

Appendix A Supplementary data associated with this article can be found in the online version at doi:10.1016/j.semcdb.2025.103630.
