# Supplementary material for: Evolvability in vertebrate segmentation
Source: Semin Cell Dev Biol. 2025 Oct;174:None. doi: 10.1016/j.semcdb.2025.103630 (PMC13507662; doi:10.1016/j.semcdb.2025.103630)
Supplement: Supplementary file 1 [file mmc1.docx]

# Supplementary Material

| Species | Class | Period (mins) | Total somites | Phylotypic stage (hpf) | Temperature | Method | Source(s) |
| --- | --- | --- | --- | --- | --- | --- | --- |
| *Amphiprion ocellaris* | Actinopterygii | 35 | 22 | 45 | *o*  26 C | Reported | [149] |
| *Anguilla japonica* | Actinopterygii | 37.5 | 114 | 85 | *o*  22 C | Reported | [150], † |
| *Astatotilapia calliptera* | Actinopterygii | 73.7 | 30 | 72 | *o*  28 C | Reported | [151] |
| *Astyanax mexicanus* | Actinopterygii | 20 | 40 | — | *o*  23 C | Reported | [97] |
| *Crassius auratus* | Actinopterygii | 30 | 35 | 26 | *o*  24 C | Reported | [152] |
| *Clupea harengus* | Actinopterygii | 52 | 61 | — | *o*  15 C | Reported | [134] |
| *Clupea pallasii* | Actinopterygii | 72.8 | 54 | 139 | *o*  9.6 C | Inferred | [153] |
| *Danio rerio* | Actinopterygii | 25.1 | 32 | 24 | *o*  27 C | Reported | [25] |
| *Dicentrarchus labrax* | Actinopterygii | 109 | 28 | 72 | *o*  15 C | Inferred | [154] |
| *Gadus morhua* | Actinopterygii | 207 | 52 | 250 | *o*  7 C | Reported | [155, 156] |
| *Leucopsarion petersii* | Actinopterygii | 187 | 36 | — | *o*  19 C | Inferred | [157] |
| *Luciobrama macrocephalus* | Actinopterygii | 22 | 55 | 60 | *o*  21 C | Inferred | [158] |
| *Misgurnus anguillicaudatus* | Actinopterygii | 36 | 50 | — | *o*  20 C | Reported | [159] |
| *Muraenesox cinereus* | Actinopterygii | 31.6 | 149 | — | *o*  24 C | Reported | [150] |
| *Oryzias latipes* | Actinopterygii | 57.5 | 35 | 101 | *o*  27 C | Reported | [138, 160] |
| *Rhamphochromis* sp. ‘chilingali’ | Actinopterygii | 67.7 | 36 | 72 | *o*  28 C | Reported | [151] |
| *Salmo salar* | Actinopterygii | 155.8 | 67 | 228.5 | *o*  13 C | Reported | [98] |
| *Anas platyrhynchos* | Aves | 80.4 | 50 | 96 | *o*  38 C | Inferred | [161] |
| *Columba livia* | Aves | 59.4 | 44 | 96 | *o*  36 C | Inferred | [162] |
| *Gallus gallus* | Aves | 90 | 52 | 84 | *o*  38 C | Reported | [19, 163] |
| *Lonchura striata* | Aves | 87.7 | 45 | 132 | *o*  38 C | Inferred | [164] |
| *Pygoscelis adeliae* | Aves | 187 | 50 | 240 | *o*  34 C | Inferred | [165] |
| *Chiloscyllium punctatum* | Chondrichthyes | 228.5 | 80 | 336 | *o*  25 C | Inferred | [166] |
| *Petromyzon marinus* | Hyperoartia | 241 | 101 | 384 | *o*  18 C | Inferred | [167] |
| *Bos taurus* | Mammalia | 310 | 56 | 626 | *o*  37 C | Inferred | [24, 168] |
| *Callithrix jacchus* | Mammalia | 778 | 56 | 1440 | *o*  37 C | Inferred | [24, 169,  170] |
| *Ceratotherium simum* | Mammalia | 236 | 44 | — | *o*  37 C | Inferred | [24, 171] |
| *Mus musculus* | Mammalia | 120 | 65 | 216 | *o*  37 C | Reported | [172] |
| *Oryctolagus cuniculus* | Mammalia | 143 | 45 | 252 | *o*  37 C | Inferred | [24, 173,  174] |
| *Aspidoscelis uniparens* | Reptilia | 240 | 90 | — | *o*  28 C | Reported | [23] |
| *Chelonia mydas* | Reptilia | 395.2 | 43 | 168 | — | Inferred | [175] |
| *Chelydra serpentina* | Reptilia | 1123.6 | 31 | 600 | *o*  20 C | Inferred | [96] |
| *Elaphe quadrivirgata* | Reptilia | 86.4 | 297 | 264 | *o*  30 C | Reported | [176] |
| *Emydura subglobosa* | Reptilia | 284 | 39 | 144 | *o*  30 C | Inferred | [177] |
| *Lamprophis fuliginosus* | Reptilia | 60 | 300 | — | *o*  28 C | Reported | [23] |
| *Pantherophis guttatus* | Reptilia | 100 | 315 | 768 | *o*  28 C | Reported | [23] |

^Table 1:^ **Somitogenesis period at a given temperature, the time to phylotypic stage, and total somite number, across species.** ^For^

each species the periodicity of somitogenesis, the time elapsed before one somite is formed, was either taken from a previously published value (‘reported’), or was inferred by linear regression of somites over time from published data (‘inferred’). The time taken to reach phylotypic stage, defined as the presence of pharyngeal arches, was not always reported and so in some cases this data is missing.^†^A Seleit *pers. comm.*
